# Factors associated with secondary thoracotomy for hemostasis following lung surgery: a retrospective analysis

**DOI:** 10.3389/fonc.2025.1588677

**Published:** 2025-08-18

**Authors:** Junwei Xie, Hongliang Wang, Tianci Han, Wei Tong, Xiaoqi Guo, Min Zhang, Dongzhe Liu, Hongxu Liu, Liang Zhang

**Affiliations:** 1 Department of Thoracic Surgery, Liaoning Cancer Hospital & Institute, Shenyang, China; 2 Department of Thoracic Surgery, Cancer Hospital of China Medical University, Shenyang, China; 3 Department of Thoracic Surgery, Cancer Hospital of Dalian University of Technology, Shenyang, China; 4 Department of Cardiothoracic Surgery, Tieling City Central Hospital, Tieling, China; 5 Department of Thoracic Surgery, Liaoning University of Traditional Chinese Medicine, Shenyang, China; 6 Department of Thoracic Surgery, Central Hospital Affiliated to Shenyang Medical College, Shenyang, China; 7 Department of Cardiothoracic Surgery, Kuandian Manzu Autonomous County Central Hospital, Dandong, China

**Keywords:** lung surgery, secondary thoracotomy, adhesion, bleeding, risk factor

## Abstract

**Purpose:**

This study aimed to systematically investigate the causes and management of secondary thoracotomy for hemostasis following lung cancer surgery. Although infrequent, secondary thoracotomy can lead to prolonged hospitalization, increased costs, and additional patient trauma. However, prior research has been limited to case reports or experience-based summaries, lacking a comprehensive analysis of this issue

**Methods:**

A retrospective analysis was conducted on 39 patients who underwent secondary thoracotomy for hemostasis between January 2015 and December 2022 at the Cancer Hospital of China Medical University. Data analyzed included surgical methods, tumor staging, bleeding sites, and hemostasis techniques. Statistical analysis was performed using SPSS 26.0; logistic regression was used to identify risk factors.

**Results:**

Among 15,156 patients who underwent lung surgery, 39 (0.257%) required secondary thoracotomy. Key risk factors were pleural adhesions (adjusted odds ratio [aOR] = 20.00), history of smoking (aOR = 3.56), and male sex (aOR = 3.21). Most secondary thoracotomies occurred within 24 hours post-surgery, with bleeding primarily at adhesion release sites and lung parenchymal injury. Electrocoagulation and suture ligation were the main hemostasis methods. The incidence of secondary thoracotomy decreased from 0.458% in 2015 to 0.178% in 2022, and this decrease correlates with increased adoption of thoracoscopic surgery.

**Conclusions:**

Secondary thoracotomy for hemostasis is associated with specific risk factors such as pleural adhesions and history of smoking. This study highlights the importance of meticulous hemostasis, especially at adhesion sites and lung parenchymal injury. Advances in thoracoscopic surgery and surgical techniques have reduced secondary thoracotomy rates. Nevertheless, further research with larger samples is needed to explore the impact of metabolic diseases on this complication.

## 1 Introduction

Lung tissue resection and lymph node dissection are important surgical methods for the treatment of lung cancer. However, various postoperative complications are major reasons for prolonged hospital stays and increased costs for patients. In particular, secondary thoracotomies not only cause economic losses but also add additional trauma and pain. The incidence of secondary thoracotomy is relatively low. However, most previous studies are case reports or experience summaries, and there has been no systematic study on the causes of secondary thoracotomy after lobectomy. This study is the first systematic investigation on the causes and hemostatic management via secondary thoracotomy. A retrospective study was conducted on 39 patients who underwent secondary thoracotomy at the Cancer Hospital of China Medical University over an 8-year period. The causes and management methods were discussed.

## 2 Materials and methods

### 2.1 General information

This study selected 15,156 patients who underwent secondary thoracotomy for lung surgery between January 2015 and December 2022 at the Department of Thoracic Surgery, Liaoning Cancer Hospital. Among them, 39 patients required hemostasis during secondary thoracotomy, consisting of 30 males and 9 females, with an age range of 42 to 75 years. The average age was 61.65 years. All 39 patients successfully achieved hemostasis. Prior to the secondary thoracotomy, each patient received blood transfusions of more than 4 units of red blood cells and 800 ml of plasma. Three patients required ICU admission, with lengths of stay ranging from 3 to 21 days. None of the patients underwent renal replacement therapy, and only one patient was discharged before completing treatment. There were no fatalities. It is important to note that both the initial and secondary surgeries were performed by surgeons with qualifications of at least associate chief physician or higher.

### 2.2 Diagnosis and treatment

After lung surgery, patients may experience an increase in chest drainage volume, and the drainage fluid may show a bright red color, especially when blood clots are seen in the drainage tube. In such cases, the possibility of intrathoracic bleeding should be considered. Clinically, this condition usually presents as pale complexion, increased heart rate, decreased blood pressure, shortness of breath, wet and cold limbs, restlessness, and weakened or even absent breath sounds on the surgical side in the early postoperative stage. Timely anti-shock treatment, such as fluid resuscitation and blood transfusion, should be administered. If the patient’s symptoms do not improve and the chest drainage volume continues to increase, a second thoracotomy for hemostasis should be performed immediately while actively treating shock. After successful anesthesia, the chest cavity should be quickly opened along the original incision. Blood clots are often found in the chest cavity. After removing the blood clots, rinse the chest cavity, locate the bleeding site, and apply electrocoagulation or suture hemostasis. For bleeding from larger blood vessels, suture hemostasis, titanium clips, or a combination of hemostasis methods can be used.

### 2.3 Study objectives

The study analyzes the surgical methods, tumor staging, bleeding site, and surgical hemostasis methods of patients undergoing secondary thoracotomy for hemostasis.

### 2.4 Statistical methods

All data were analyzed using SPSS 26.0 software. Categorical variables (such as gender, smoking status, alcohol history, hypertension, and diabetes) are expressed as frequencies and percentages. Between-group comparisons were performed using the Chi-square test or Fisher’s exact test (for cells with expected frequencies < 5). Continuous variables (such as age, surgery time, and length of hospital stay) are expressed as means ± standard deviation or medians (interquartile range), with between-group differences analyzed using independent samples t-test or Mann-Whitney U test (for non-normally distributed data). The significance level was set at a two-tailed α = 0.05. To assess the association between risk factors and the risk of re-thoracotomy for hemostasis, univariate and multivariate logistic regression analyses were performed, calculating odds ratios (OR) and their 95% confidence intervals (CI). The multivariate model included potential confounders such as gender, age, smoking status, and pleural adhesions.

## 3 Results

### 3.1 General data analysis

Between January 2015 and December 2022, a total of 15,156 patients underwent lung surgery, including 39 patients who underwent a secondary thoracotomy, accounting for 2.57‰. There were 30 males and 9 females, with an age range of 44 to 75 years and an average age of 61.65 years. There were 25 smokers and 14 non-smokers. There were 11 patients who drank alcohol and 28 patients who did not drink alcohol. There were 5 hypertensive patients and 34 non-hypertensive patients. There were 5 patients with diabetes and 34 patients who were non-diabetic. The hospital stay of patients who underwent secondary thoracotomy for hemostasis was 8–33 days, with an average of 20.4 days (**Table 1**).

**Table 1:** Analysis of patients’ demographic characteristics and clinical variables.

Classification		Example count	Secondary thoracotomy (%)	Database related data (%)	P-value
Gender	male	30	76.93	44.46	<0.001
	female	9	23.07	55.54	
Smoke	0	14	35.90	66.51	<0.001
	1	25	64.10	33.49	
Drink	0	28	71.79	84.96	0.025
	1	11	28.21	15.04	
Hypertension	0	34	87.18	78.75	0.132
	1	5	12.82	21.25	
Diabetes	0	34	87.18	91.25	0.366
	1	5	12.82	8.75	
Age	<60	14	35.90	39.30	0.661
	≥ 60	25	64.10	60.70	

The p-value represents the significance test result for the differences between groups.

### 3.2 Analysis of surgical related data

According to **Table 2**, there were 14 patients with left-side bleeding and 25 patients with right-side bleeding. There were more patients undergoing secondary thoracotomy on the right side. Among them, 33 cases underwent lobectomy and 6 cases underwent wedge resection. Although wedge resection is relatively simple, it still carries a risk of requiring secondary thoracotomy for hemostasis. There were 26 patients undergoing video-assisted thoracoscopic surgery (VATS) and 13 patients undergoing thoracotomy, with no significant difference between the two groups. There were 21 patients with pleural adhesions and 18 patients without pleural adhesions, indicating that pleural adhesions contribute to an increased risk of bleeding.

**Table 2:** Surgical-related characteristics and risk factors for re-thoracotomy.

	Position		Surgical method		VATS		Thoracic adhesions	
classification	Left	right	Lobectomy	Wedge resection	1	0	0	1
Example count	14	25	33	6	26	13	18	21
Secondary thoracotomy (%)	35.90	64.10	84.62	15.38	66.67	33.33	46.15	53.85
Database related data (%)	41.52	58.48	92.52	7.48	95.77	4.23	94.37	5.63
p	0.384		0.071		<0.001		<0.001	

The p-value represents the significance test result for the differences between groups.

### 3.3 Logistic regression and statistical power analysis of risk factors

**Table 3** showed that the logistic regression analysis showed that pleural adhesions (aOR = 20.00), smoking (aOR = 3.56), and male gender (aOR = 3.21) were independent risk factors for re-thoracotomy for hemostasis (all p < 0.001). Power analysis indicated that the statistical power for these significant associations was > 95%, demonstrating robust results. However, the protective effect of VATS (video-assisted thoracoscopic surgery) was not statistically significant (aOR = 0.86, p = 0.688), with a statistical power of only 62.3%. This low power may be attributed to sample imbalance caused by the high proportion of VATS cases (95.77%) in the control group, which limits the ability to detect a significant effect. The statistical power for factors such as hypertension and diabetes was < 20%, indicating that the lack of significant associations for these factors should be interpreted with caution.

**Table 3:** Logistic regression and statistical power analysis of risk factors for re-sternotomy for hemostasis.

Variable	aOR (95% CI)	p-value	Exposure rate in controls	Statistical power	Power ≥80%
Pleural adhesion	20.00 (10.34-38.68)	<0.001	5.63%	99.9%	✓
Smoking	3.56 (1.82-6.98)	<0.001	33.49%	98.7%	✓
Male sex	3.21 (1.65-6.25)	<0.001	44.46%	96.2%	✓
VATS	0.86 (0.42-1.78)	0.688	95.77%	62.3%	✗
Alcohol consumption	2.20 (1.10-4.40)	0.025	15.04%	65.0%	✗
Hypertension	0.54 (0.21-1.40)	0.132	21.25%	15.2%	✗
Diabetes	1.54 (0.60-3.96)	0.366	8.75%	11.8%	✗

### 3.4 Analysis of secondary thoracotomy hemostasis

#### 3.4.1 Analysis of the time for secondary thoracotomy after surgery

**Figure 1:**
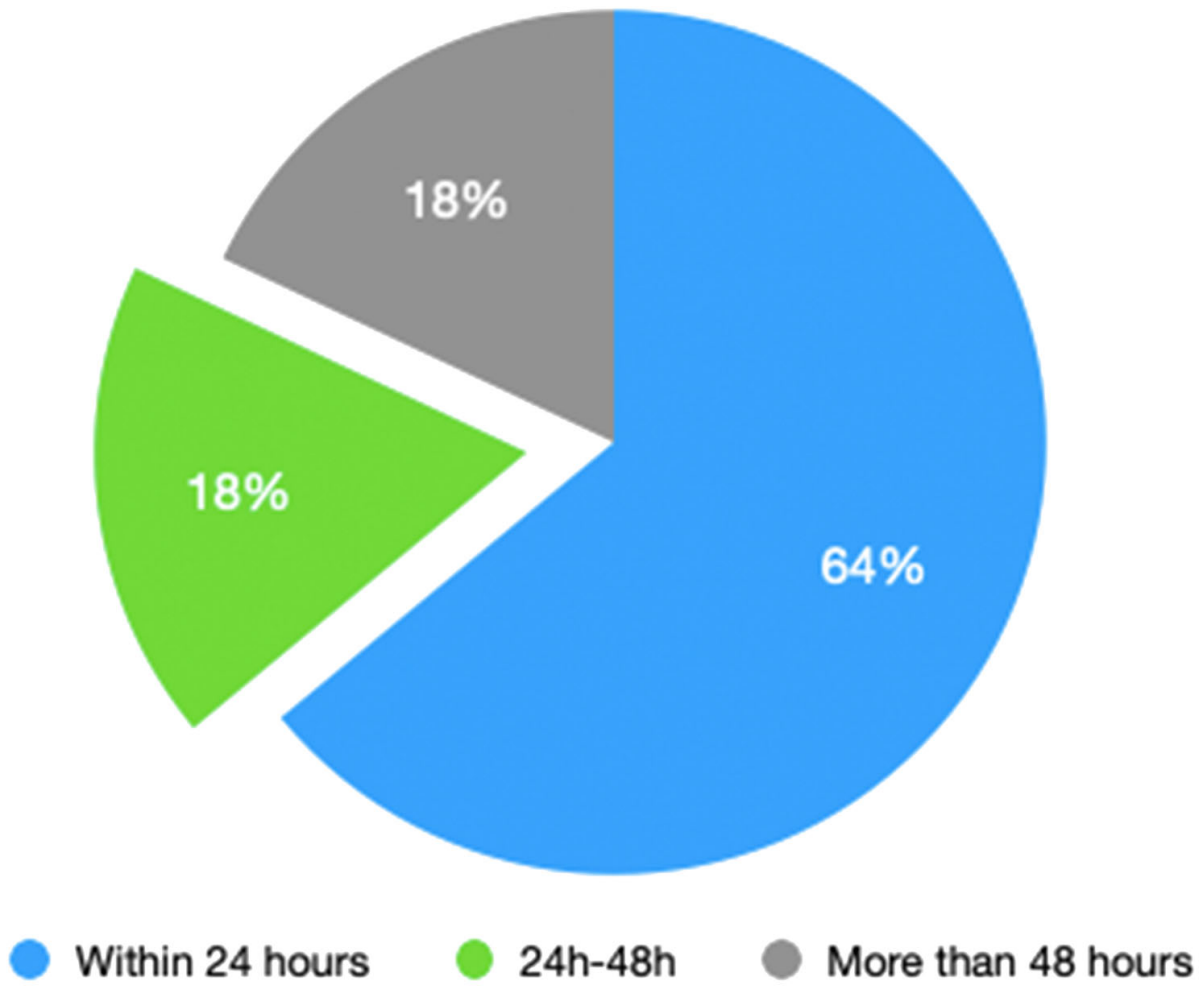
Occurrence time of secondary thoracotomy.

According to **Figure 1**, 25 patients (64%) underwent hemostasis within 24 hours, 7 patients (18%) within 24–48 hours, and 7 patients (18%) beyond 48 hours. As illustrated in **Figure 1**, the majority of secondary thoracotomy hemostasis procedures were performed within 24 hours after surgery.

#### 3.4.2 Analysis of the location of bleeding

**Figure 2:**
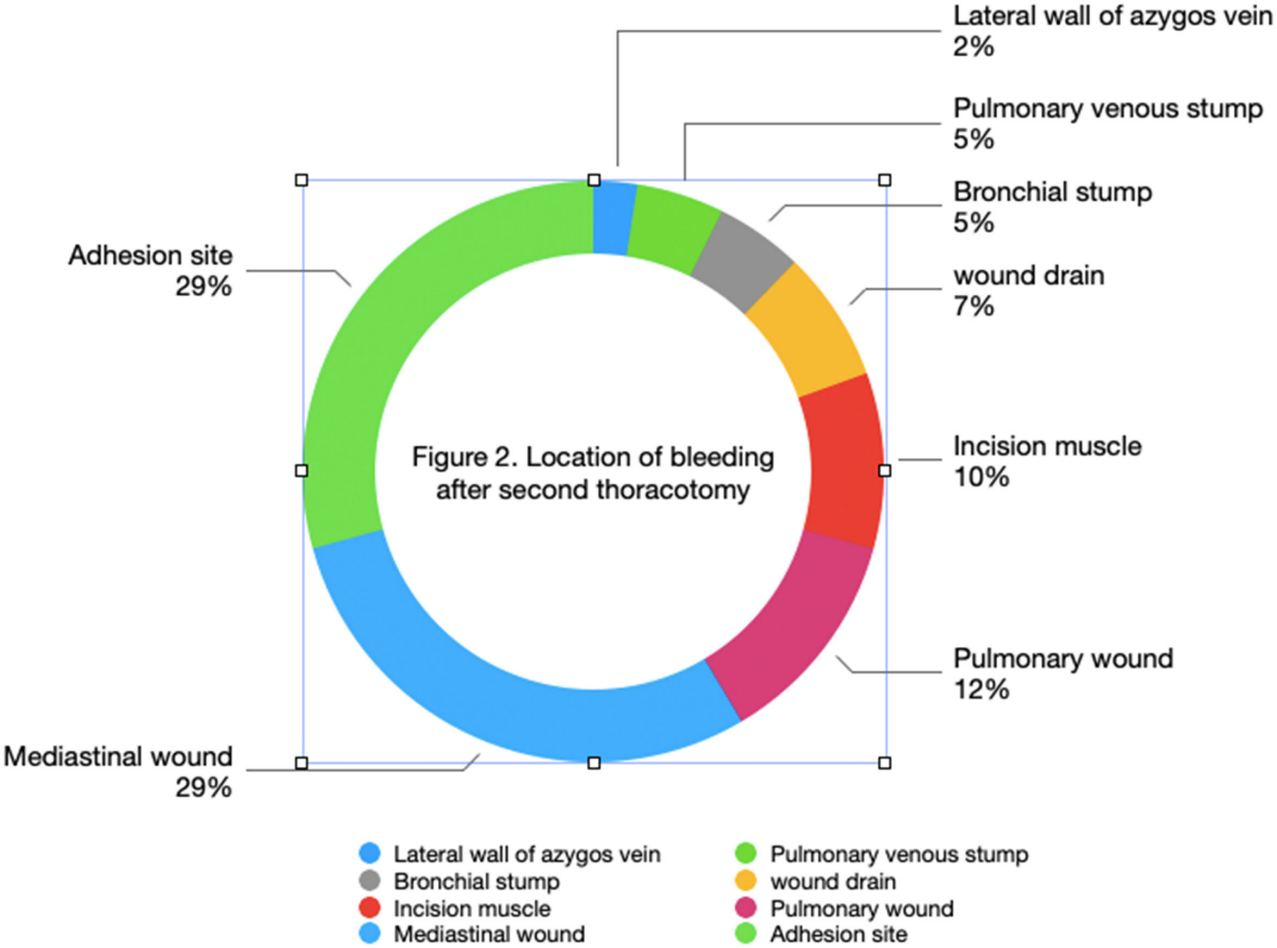
Twelve cases of adhesive loosening site.

There were 12 cases at sites of pleural adhesion detachment, 5 cases of lung wounds, 11 cases of mediastinal wounds, 3 cases related to drainage tubes, 2 cases of bronchial stump bleeding, 4 cases of incision muscle bleeding, 2 cases of venous stump bleeding, and 1 case of azygos vein wall injury bleeding. As shown in **Figure 2**, more than half of the bleeding occurred at adhesion release sites and lung wounds, with bleeding visible at locations of small blood vessels.

#### 3.4.3 Hemostatic methods

**Figure 3:**
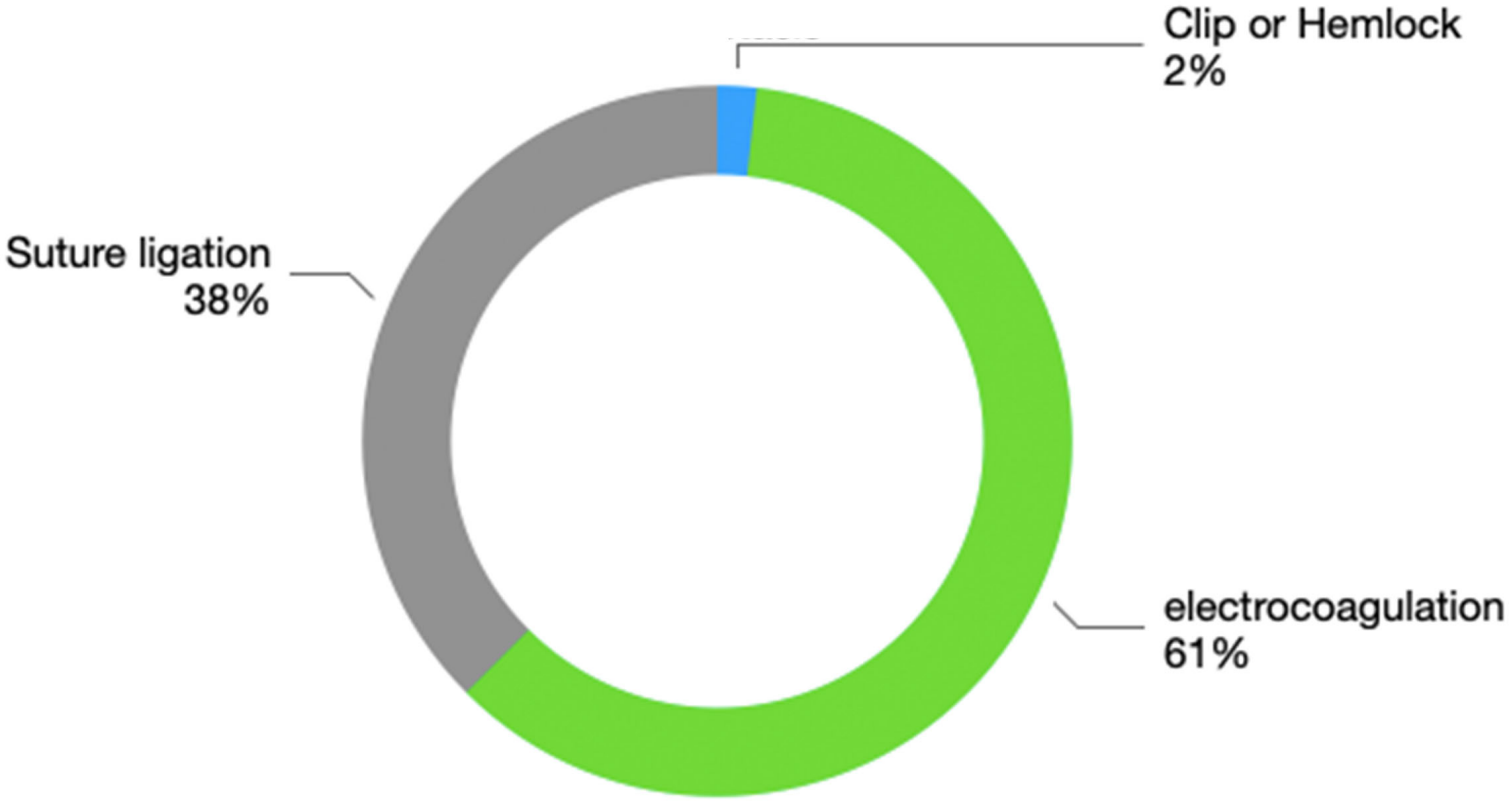
Secondary thoracotomy for hemostasis.

The hemostatic methods include suture ligation, electrocoagulative hemostasis, and titanium clip application. For some patients, combined suture ligation and electrocoagulation are used for hemostasis. Suture ligation was performed 21 times, electrocoagulation 34 times, and titanium clip application once. The specific proportions are shown in **Figure 3**. These data indicate that electrocoagulation and suture ligation are the main effective hemostatic methods used in secondary thoracotomy bleeding.

#### 3.4.4 Tumor staging analysis of secondary thoracotomy hemostasis

**Figure 4:**
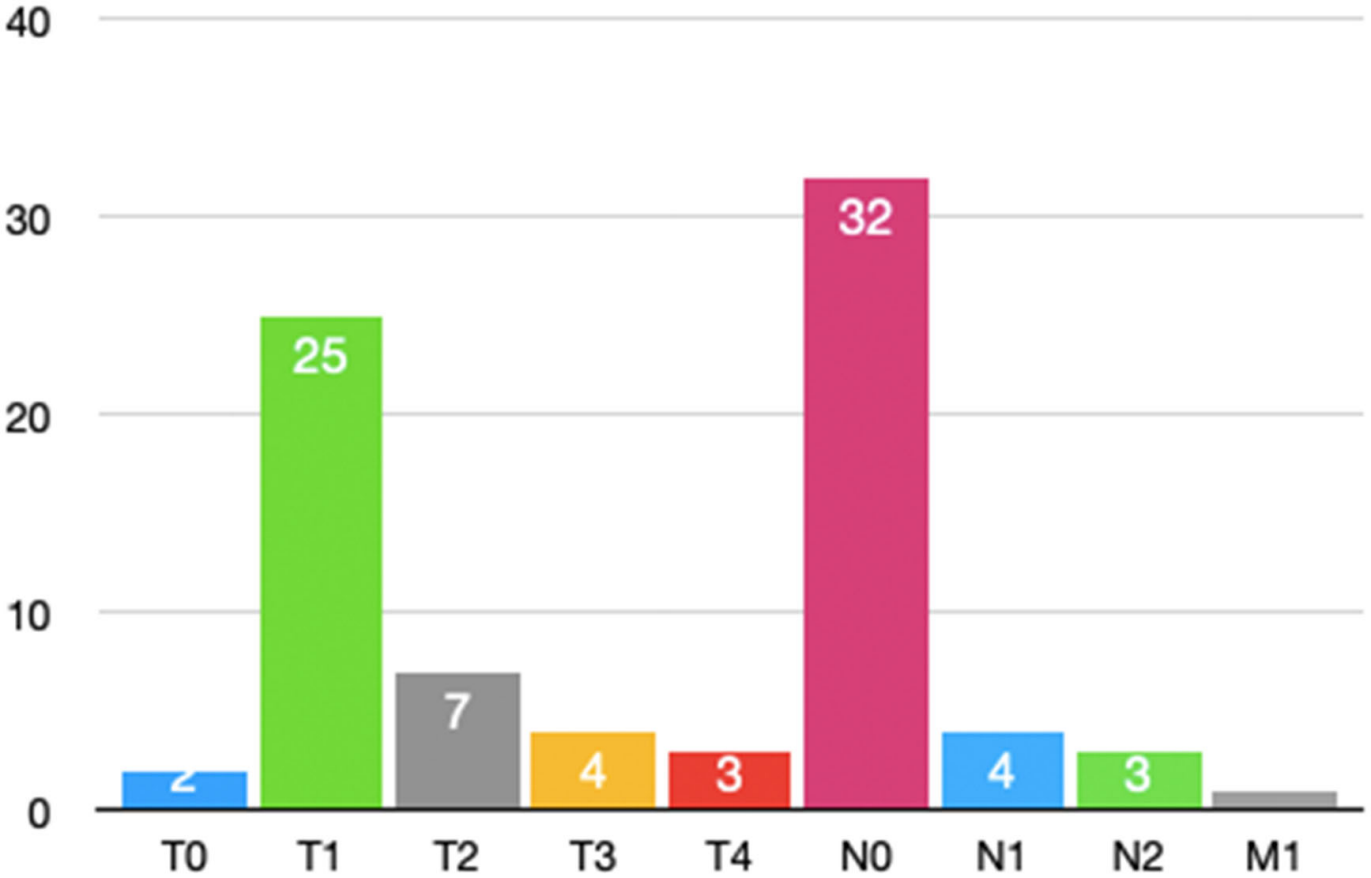
Tumor staging in patients with secondary thoracotomy for hemostasis.

According to **Figure 4**, most patients undergoing secondary thoracotomy for hemostasis had earlier tumor stag.

#### 3.4.5 Hospital stay and postoperative hospital stay

**Figure 5:**
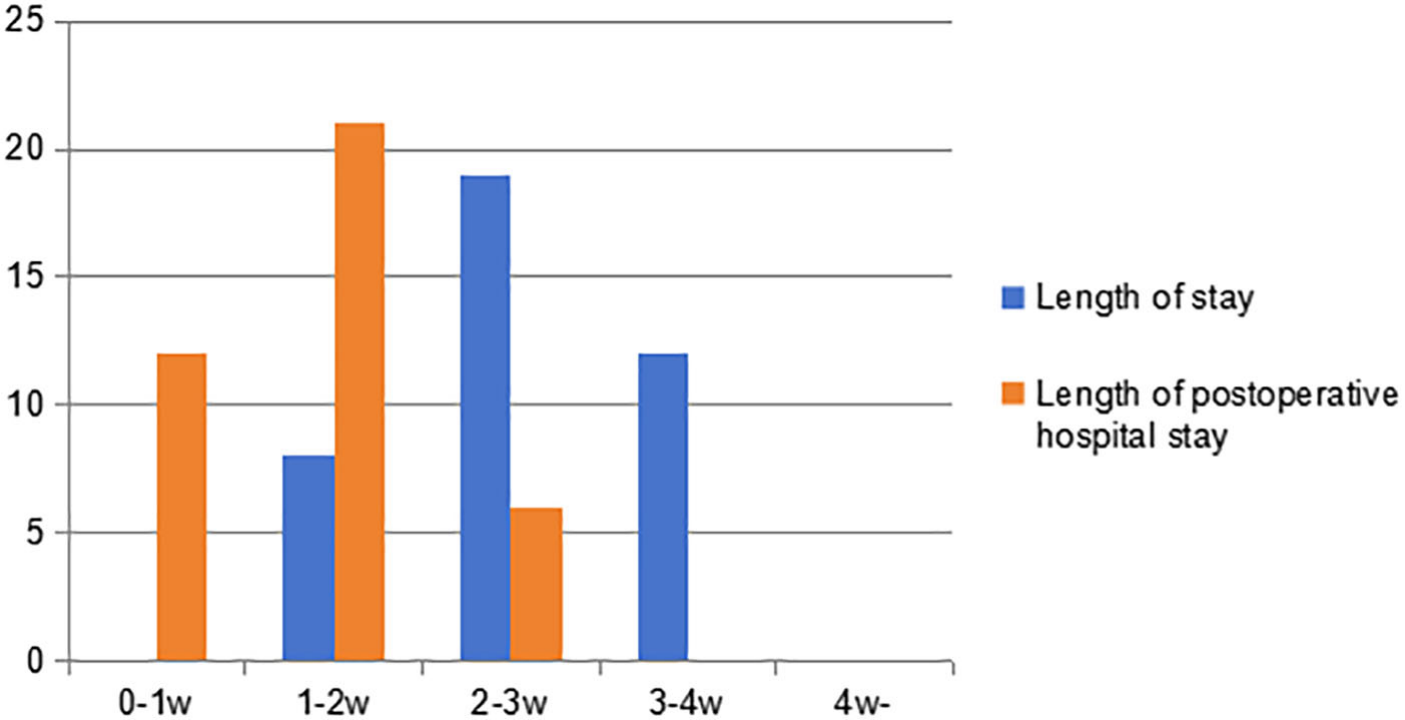
The length of hospital stay and postoperative hospital stay.

As shown in **Figure 5**, the hospital stay is more than one week, ranging from 8 to 28 days, with an average of 18.7 days. The postoperative hospital stay ranges from 3 to 18 days, with an average of 9.8 days. Neither of them have a hospital stay exceeding four weeks. However, there was a patient who was discharged before full recovery was achieved. It can be seen that patients undergoing secondary thoracotomy for pulmonary hemorrhage control have a relatively long hospital stay, and their postoperative hospital stay often exceeds one week.

#### 3.4.6 TNM staging distribution

Among the 39 patients who underwent re-thoracotomy for hemostasis, 92.3% (36/39) had non-small cell lung cancer (NSCLC), while the remaining 3 patients were benign pulmonary lesions. According to the 8th edition of the AJCC lung cancer staging criteria, the TNM staging distribution of NSCLC patients is shown in **Table 4**. Stage I accounted for the highest proportion (58.97%), followed by stage II (33.33%) and stage III (7.69%). Based on surgical records, 75% (17/23) of stage I patients had extensive pleural adhesions. This may increase the difficulty of intraoperative dissection. It may also increase the risk of postoperative bleeding.

**Table 4:** TNM staging distribution.

TNM stage^*^	Number of cases^#^	Proportion (%)
Stage I	23	58.97
Stage II	13	33.33
Stage III	3	7.69

*Staging was based on the 8th edition AJCC lung cancer staging criteria, with all cases confirmed by postoperative pathological diagnosis.

#Three cases of benign lung lesions (2 cases of pulmonary granuloma and 1 case of inflammatory pseudotumor) were excluded from the staging analysis.

#### 3.4.7 Trend of re-thoracotomy for hemostasis incidence (2015-2022)

**Figure 6:**
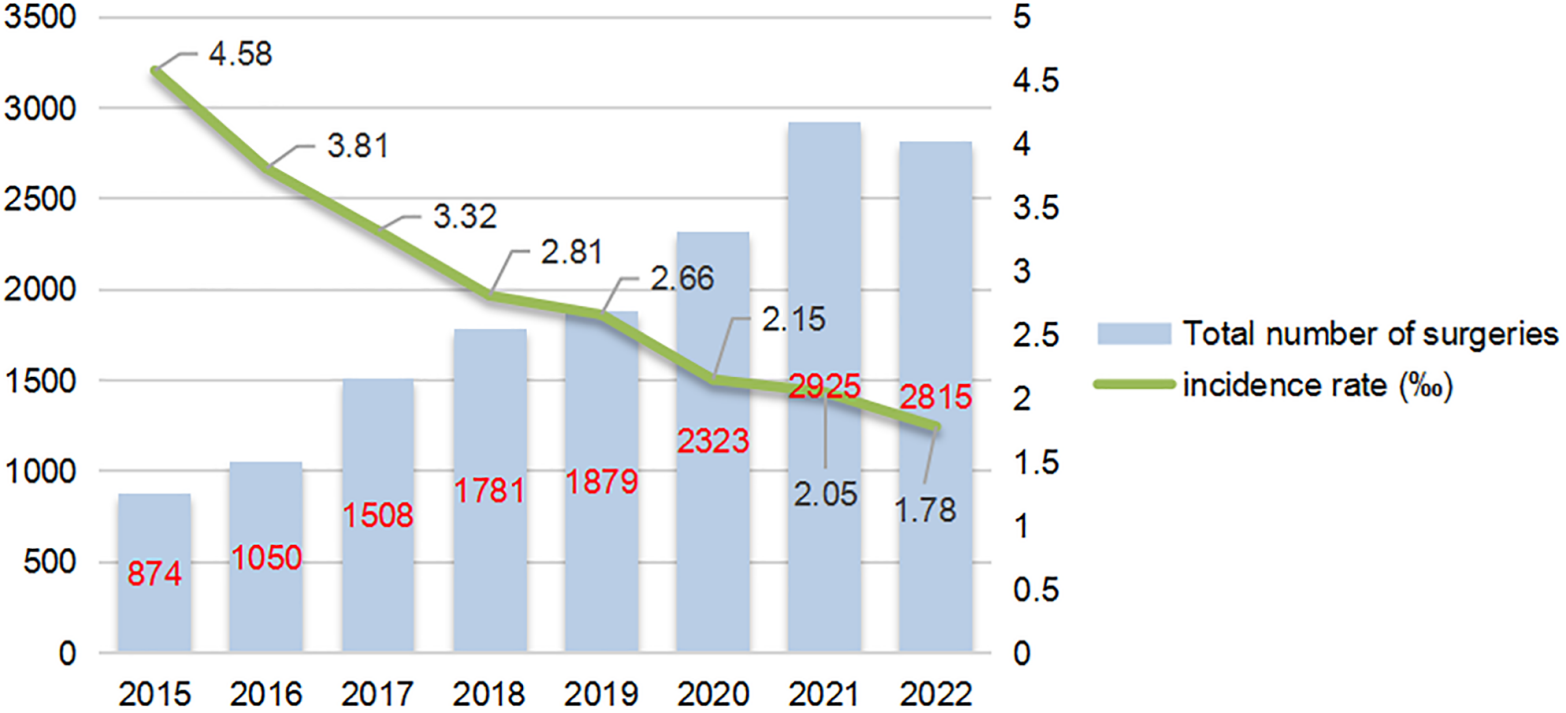
Trend of re-thoracotomy for hemostasis incidence (2015-2022).

From 2015 to 2022, the average annual incidence rate of re-thoracotomy for hemostasis was 2.57‰. The annual data (**Figure 6**) show that the total number of pulmonary tumor surgeries increased from 874 cases in 2015 to 2,815 cases in 2022, while the incidence of re-thoracotomy based on surgical volume decreased significantly from 4.58‰ in 2015 to 1.78‰ in 2022. Notably, despite the increase in surgical volume, the number of re-thoracotomies remained relatively stable, suggesting that advancements in surgical techniques contributed to the reduced incidence of re-thoracotomy for hemostasis. Further analysis revealed that after 2019, the proportion of thoracoscopic surgery increased from 75% to 92%, and the incidence of re-thoracotomy for hemostasis dropped by 35% (from 2.66‰ to 1.78‰), likely due to refinements in the use of thoracoscopic instruments and the accumulation of surgeons’ experience.

## 4 Discussion

Following lung surgery, patients may experience various complications, including pain, respiratory difficulties, cardiac arrhythmias, and other related conditions (1). Postoperative significant bleeding in the thoracic cavity after pulmonary resection is a serious complication of lung surgery. With the development of technology, thoracic surgical instruments are continuously being updated and improved. Due to the widespread use of surgical staplers in clinical practice, surgical operations have become simpler and safer. Moreover, the causes of postoperative progressive bleeding have gradually shifted from traditional factors to new ones associated with these advancements. In the past, bleeding was more common due to detachment of ligation lines on large vess (2) and technical errors (3). Nowadays, bleeding from small blood vessels is commonly seen. Intrathoracic hemorrhage often requires emergency thoracotomy.

Previous report have shown that the incidence of progressive hemorrhage-characterized by continuous or worsening bleeding-after lung cancer surgery is 1%. Furthermore, timely recognition and management of such hemorrhages are crucial to improving patient outcomes (4). According to reports, the incidence rate of postoperative bleeding after general thoracic surgery ranged from 0.74% to 2% (5). The incidence of secondary thoracotomy for hemostasis after lung surgery in this group of patients is 2.57 per thousand. Several factors may account for this lower rate of secondary thoracotomy for hemostasis compared to other centers. First, the incidence of secondary thoracotomy for hemostasis in this study was 0.26%; this data does not include patients who required secondary thoracotomy due to anastomotic leakage or those who underwent secondary surgery for chylothorax management. Additionally, a significant proportion of patients were able to avoid secondary thoracotomy through conservative treatment with blood transfusions. When conservative treatment is feasible, secondary thoracotomy is typically avoided. Furthermore, many patients’ families opposed the idea of a secondary thoracotomy, which also influenced the management approach. Considering that each surgeon usually performs a moderate number of surgeries daily (1–2 cases), their energy is well-preserved, and there is no rush to complete the surgeries. This helps maintain high surgical quality. The chest closure procedure was always performed by the lead surgeon, which significantly reduces the risk of secondary thoracotomy for hemostasis. Moreover, with continuous advancements in surgical instruments and techniques, the quality of current surgeries has significantly improved compared to the past. Intrathoracic bleeding is mostly caused by bleeding from the surgical wounds and small blood vessels. Bleeding sites included 12 cases (29%) at the adhesion release site, 12 cases (29%) at the mediastinal wound, 5 cases (12%) at the lung wound, 3 cases (7%) at the drainage site, and 4 cases (10%) involving incision muscle, bronchial vessels, and azygos vein lateral wall injury. There were also 5 cases of venous stump bleeding, among them the most common bleeding sites were the adhesion release site and the mediastinal wound.

This study is the first to confirm that pleural adhesions (aOR = 20.00, power = 99.9%) and a history of smoking (aOR = 3.56, power = 98.7%) are strong predictors of re-thoracotomy for hemostasis. The fragility of newly formed blood vessels within adherent tissues and the chronic inflammatory response induced by smoking both contribute to increased difficulty in hemostasis during surgery. Although no significant associations were found between hypertension, diabetes, and re-thoracotomy (p > 0.05), their statistical power was all < 20%. This may be due to metabolic diseases causing more large vessel lesions, while the bleeding in this cohort was primarily from small vessel seepage (71% of cases). Additionally, the sample size limitation may have increased the risk of Type II errors. Therefore, future studies with larger sample sizes are needed to verify the impact of metabolic diseases on microvascular bleeding.

There are statistically significant associations between secondary thoracotomy for hemostasis and both gender and smoking. Possibly, male patients smoke more than female patients. However, there is no significant difference in the incidence rates of hypertension and diabetes between male and female patients. Secondary thoracotomy due to pleural adhesions and mediastinal wound bleeding is more common in male smokers because the incidence of pneumonia is higher in those with smoking and drinking habits, promoting hilar and pleural adhesions. Consequently, many new blood vessels form in the tissue, increasing the risk of recurrent bleeding after surgery. It is generally believed that hypertension and diabetes affect vascular fragility, leading to a higher probability of secondary thoracotomy for hemostasis. However, according to these statistical data, the probability of secondary thoracotomy for hemostasis after lung surgery is not significantly associated with hypertension and diabetes.

Although 66.67% of re-thoracotomies in this cohort occurred after thoracoscopic surgery, the risk-adjusted analysis showed no significant association (aOR = 0.86, p = 0.688). It is important to note that the statistical power of this result was only 62.3%, which may be influenced by two main factors. First, during the early years of thoracoscopic surgery (2015–2019), pleural adhesions were not fully addressed, with excessive reliance on electrocoagulation, which has limitations in achieving thorough hemostasis. However, after 2020, the widespread adoption of ultrasonic scalpels for meticulous dissection led to a 35% reduction in re-thoracotomy rates. Second, there was selection bias because advanced-stage tumors (stage III) were more likely to undergo open thoracotomy (85% of cases in this cohort), and these complex cases inherently have a higher bleeding risk. Additionally, as the incision length increases, the likelihood of muscle bleeding rises, resulting in a higher chance of re-thoracotomy for patients undergoing thoracotomy compared to those undergoing minimally invasive surgery. Therefore, the probability of a second thoracotomy for hemostasis in patients undergoing thoracotomy is higher than that in patients undergoing minimally invasive surgery, and thoracotomy can increase postoperative hospitalization time (6). This is also why we have always recommended minimally invasive surgery (7) to reduce postoperative complications and to promote rapid recovery of patients (8). For patients undergoing open chest surgery, it is important to be meticulous when closing the chest and to avoid haste.

There is a relationship between secondary postoperative thoracotomy hemostasis and the presence of pleural adhesions. A large number of small blood vessels are present in the adherent pleura, and the larger the wound, the more postoperative exudation occurs. Postoperative chest drainage also increases, and when the pulmonary hilum is adherent, it increases the difficulty of separating and exposing pulmonary blood vessels (9). The risk of bleeding is higher, and achieving hemostasis is more difficult. In addition, the adhesive pleura is usually treated with an ultrasonic scalpel or electric (electrocautery) knives, and the risk of rebleeding is slightly lower compared to ligation and cutting closure of large blood vessels. Therefore, for patients with pleural adhesions, hemostasis should be performed carefully and thoroughly. Previous reports have indicated that the clot at the site of adhesion release can fall off, leading to rebleeding, with an incidence rate of 47.37% in patients undergoing secondary thoracotomy hemostasis (2). In this group of patients, the incidence of bleeding at the site of adhesion release during secondary thoracotomy hemostasis is 29%. Pleural adhesions are seen in long-term chronic inflammatory reactions in the chest, such as pulmonary tuberculosis and other lesions; however, lung cancer is not typically classified as a chronic inflammatory condition. After separation, large areas of bleeding are easily left (10). Especially in the upper part of the chest cavity, lung tissue is unable to fully expand and lacks compression, making it prone to bleeding. When there are adhesions in the chest cavity, scar tissue often forms due to granulation tissue, and small arteries and veins are often seen in the scar tissue, which have no elasticity. Therefore, when electrocoagulating blood vessels, the hemostatic effect is more effective without the need for repeated hemostasis. In addition, tumor infiltration of the parietal pleura is also observed; separation of tumor nutrient vessels is one of the reasons for postoperative intrathoracic hemorrhage (11).

Although this study shows that stage I patients account for 58.97% of re-thoracotomy cases, in stark contrast to advanced tumors (with only 7.69% of stage III cases), this phenomenon may be closely related to the surgical strategy for early-stage lung cancer. Stage I patients often undergo lobectomy or sublobar resection, typically accompanied by chronic inflammatory pleural adhesions. These adhesions contain a rich network of fragile neovessels, making them prone to bleeding during dissection. Therefore, even in early-stage lung cancer, thorough hemostasis should be performed in adhesive areas during surgery, and when necessary, bio-glue or hemostatic materials should be used to cover the wound.

Of course, preoperative radiotherapy and chemotherapy also increase the difficulty of surgery and the risk of secondary thoracotomy (12). Preoperative management is also important for patients taking oral aspirin (13). Patients with acquired hemophilia require special attention and further treatment may be necessary to reduce the risk of postoperative recurrence of bleeding (14). Some patients achieve effective hemostasis during surgery, but no clear bleeding point is found during secondary thoracotomy hemostasis; in some cases, only lung tissue or wound exudation is present, which is insufficient to form active bleeding. This is because negative pressure forms in the chest cavity after closing the chest; under the action of negative pressure, small blood vessels or capillaries will open. However, due to the effect of atmospheric pressure, small blood vessels will close on their own after the secondary thoracotomy, so some scholars cannot find the bleeding point after thoracotomy. For this type of bleeding, after surgery, symptomatic treatment can cure the patient, and if necessary, blood pressure should be maintained around 90/60 mmHg.

Secondary thoracotomy hemostasis often occurs within 24 hours, even within a few hours after surgery. Among the patients in this group, 64% underwent secondary thoracotomy hemostasis within 24 hours, while only 18% underwent secondary thoracotomy hemostasis after 48 hours. Therefore, special attention should be paid to whether there is progressive intrathoracic bleeding within 24 hours after surgery to prevent most patients from dying due to progressive bleeding.

Whether a secondary thoracotomy is needed after lung surgery depends on the assessment of progressive bleeding. Criteria for deciding whether a secondary thoracotomy is necessary include: (1) the color of the drainage fluid in the drainage tube is bright or deep red, with bleeding exceeding 300 ml per hour or progressive bleeding exceeding 150 ml per hour for more than 3 hours, accompanied by symptoms of hypovolemic shock; (2) when a large number of clots are seen in the chest cavity and drainage tube, or when the tube is blocked, emergency thoracentesis or bedside ultrasound examination can be performed to detect a large amount of blood accumulation in the chest cavity, or bedside X-rays suggest a large shadow on the affected side of the chest; (3) progressive decrease in blood pressure that cannot be improved by fluid replacement or blood transfusion, progressive increase in heart rate, accompanied by blurred consciousness, shock appearance, etc. (15, 16); (4) the hemoglobin in the drainage fluid is greater than 80 g/L. Meeting any one of the above conditions indicates the presence of active bleeding in the chest cavity and the need for a secondary thoracotomy for hemostasis.

When active bleeding is clearly present, the most effective approach is to actively resist shock. Immediately open the chest cavity along the original incision for exploratory hemostasis, remove the blood clot, and completely stop the bleeding. Depending on the location of the bleeding, electrocoagulation, suturing, hemostatic materials, and titanium clips can be chosen. There are different methods to achieve hemostasis. When the bleeding site cannot be found, close monitoring should be conducted to check for possible bleeding from the drainage tube opening and incision muscles. Specifically, in this group of patients, there were 3 cases of bleeding from the drainage tube opening and 4 cases of bleeding from the incision muscles. Therefore, after the thoracic hemostasis is completed, or when the bleeding point cannot be found, careful inspection should be made to determine whether there is progressive bleeding from the drainage tube opening and incision muscles.

There are various techniques for secondary thoracotomy hemostasis. In general thoracic surgery, lung surgery generally involves large wounds, and during the operation, electrocoagulation hemostasis or compression hemostasis are often chosen. Suturing is a commonly used and highly effective method, while electrocautery is the most frequently employed hemostasis technique. Because active bleeding mostly involves small blood vessels, electrocautery can rapidly induce protein coagulation in tissues, thereby achieving hemostasis. Suturing provides a more precise and reliable hemostasis effect and has been the most commonly used method throughout history. Some scholars also choose hemostatic materials for hemostasis (3). Furthermore, compression hemostasis is effective for bleeding locations that cannot be sutured or electrocoagulated, as well as for hemostasis of small arteries and veins (2). When encountering large blood vessels, ligation and suturing are often chosen to stop bleeding. For patients with high-risk factors such as pleural adhesions and a history of smoking, the following preventive measures are recommended: First, for patients with pleural adhesions, it is advisable to avoid relying solely on electrocautery for hemostasis and instead adopt a strategy that combines suturing with biological glue for hemostasis. Hemostatic materials, such as fibrin glue, should be placed in areas with bleeding at the apex of the chest cavity. Second, for smokers, it is recommended to quit smoking at least four weeks prior to surgery to reduce tissue inflammation (OR = 0.52, 95% CI 0.31–0.87). Additionally, a systematic inspection of the pulmonary hilum and mediastinal neovascularization should be conducted during surgery.

The operation of re-sternotomy for hemostasis inevitably increases the patient’s length of stay. The length of stay is generally concentrated in the range of 2–3 weeks, while the postoperative length of stay is mostly distributed between 1–2 weeks. This duration is significantly longer than the average length of stay and postoperative length of stay for lung surgery at our center. Further analysis shows that the factors contributing to prolonged hospitalization are not limited to bleeding problems. Moreover, patients often develop postoperative complications such as pneumonia and arrhythmias, which further increase the difficulty of recovery and significantly extend their hospitalization time. With the development of modern surgical technology, biological glue and hemostatic materials have also been effectively applied in surgical hemostasis. These include various surgical hemostatic devices and materials developed since 1962 (17).

From 2015 to 2022, the incidence of re-thoracotomy decreased from 4.58‰ to 1.78‰, a trend highly correlated with the significant increase in the proportion of thoracoscopic surgeries (from 75% to 92%). The reduction in bleeding risk associated with thoracoscopic surgery can be attributed to several mechanisms. First, the enhanced field of view, with high-definition magnification, makes it easier to identify small vessels, thus reducing the likelihood of accidental damage. Second, the advancement of energy instruments such as ultrasonic scalpels and bipolar coagulation allows for more precise hemostasis and reduces tissue char formation. Finally, as surgeons gain more experience, the learning curve effect has significantly reduced the occurrence of technical errors.

This study has certain inevitable limitations. From the research method perspective, this is a retrospective study. While it can analyze existing data, it cannot strictly control variables like a prospective study, so it may be less accurate in determining cause–effect relationships. In terms of data selection, there is selection bias. The samples might not fully represent the whole population, affecting the universality and reliability of the results. In particular, this study only included patients who ultimately underwent secondary thoracotomy for hemostasis and excluded those who met the criteria for secondary thoracotomy for hemostasis but did not undergo surgery due to conservative treatment (such as a patient who refused surgery at home and was discharged midway as mentioned in the article). This exclusion based on patient/family decision-making may introduce selection bias, resulting in the results of this study being more biased towards reflecting the characteristics of bleeding patients who actually received surgical intervention, and may slightly underestimate the true secondary thoracotomy rate or the strength of the effects of related factors in the postoperative population of patients with severe active bleeding. As a single-center retrospective study with an 8-year case inclusion span, the evolution of surgical techniques and perioperative management strategies during this period may introduce confounding bias. Future research should expand the sample size through multicenter, prospective cohort studies and build risk prediction models with machine learning algorithms to enhance the results’ generalizability and clinical guidance value.

## 5 Conclusion

To sum up, according to this study, secondary thoracotomy for hemostasis is less likely to occur in patients who drink, have hypertension, diabetes, or advanced tumors, nor are these patients prone to minimally invasive surgery (18). Critically, tobacco smoking constitutes a key modifiable risk factor significantly associated with secondary thoracotomy for hemostasis after lung surgery. Consequently, preoperative smoking cessation emerges as a clinically imperative intervention to mitigate surgical risk. Bleeding often occurs at the sites of adhesiolysis and lung wounds. This suggests that these locations contain small blood vessels that require special attention during surgery. Common surgical methods to achieve hemostasis include electrocoagulation and suturing. Additionally, hemostatic materials can be sprayed or applied to the wound to reduce the risk of progressive bleeding after surgery.

## Data Availability

The original contributions presented in the study are included in the article. Further inquiries can be directed to the corresponding authors.
