# Correction: Factors associated with secondary thoracotomy for hemostasis following lung surgery: a retrospective analysis

**DOI:** 10.3389/fonc.2026.1783337

**Published:** 2026-02-26

**Authors:** Junwei Xie, Hongliang Wang, Tianci Han, Wei Tong, Xiaoqi Guo, Min Zhang, Dongzhe Liu, Hongxu Liu, Liang Zhang

**Affiliations:** 1 Department of Thoracic Surgery, Liaoning Cancer Hospital & Institute, Shenyang, China; 2 Department of Thoracic Surgery, Cancer Hospital of China Medical University, Shenyang, China; 3 Department of Thoracic Surgery, Cancer Hospital of Dalian University of Technology, Shenyang, China; 4 Department of Cardiothoracic Surgery, Tieling City Central Hospital, Tieling, China; 5 Department of Thoracic Surgery, Liaoning University of Traditional Chinese Medicine, Shenyang, China; 6 Department of Thoracic Surgery, Central Hospital Affiliated to Shenyang Medical College, Shenyang, China; 7 Department of Cardiothoracic Surgery, Kuandian Manzu Autonomous County Central Hospital, Dandong, China

**Keywords:** lung surgery, secondary thoracotomy, adhesion, bleeding, risk factor

The order of authors in the author list of the published paper was erroneously given as:

“Junwei Xie, Hongliang Wang, Tianci Han, Wei Tong, Xiaoqi Guo, Min Zhang, Dongzhe Liu, Liang Zhang, Hongxu Liu”

The correct author list reads:

“Junwei Xie, Hongliang Wang, Tianci Han, Wei Tong, Xiaoqi Guo, Min Zhang, Dongzhe Liu, Hongxu Liu, Liang Zhang”

The original version of this article has been updated.

